# Optical neural networks: progress and challenges

**DOI:** 10.1038/s41377-024-01590-3

**Published:** 2024-09-20

**Authors:** Tingzhao Fu, Jianfa Zhang, Run Sun, Yuyao Huang, Wei Xu, Sigang Yang, Zhihong Zhu, Hongwei Chen

**Affiliations:** 1 College of Advanced Interdisciplinary Studies, National University of Defense Technology, Changsha, China; 2 Hunan Provincial Key Laboratory of Novel Nano-Optoelectronic Information Materials and Devices, National University of Defense Technology, Changsha, China; 3 Nanhu Laser Laboratory, National University of Defense Technology, Changsha, China; 4 Department of Electronic Engineering, Tsinghua University, Beijing, China; 5 Beijing National Research Center for Information Science and Technology (BNRist), Beijing, China

**Keywords:** Nanophotonics and plasmonics, Photonic devices

## Abstract

Artificial intelligence has prevailed in all trades and professions due to the assistance of big data resources, advanced algorithms, and high-performance electronic hardware. However, conventional computing hardware is inefficient at implementing complex tasks, in large part because the memory and processor in its computing architecture are separated, performing insufficiently in computing speed and energy consumption. In recent years, optical neural networks (ONNs) have made a range of research progress in optical computing due to advantages such as sub-nanosecond latency, low heat dissipation, and high parallelism. ONNs are in prospect to provide support regarding computing speed and energy consumption for the further development of artificial intelligence with a novel computing paradigm. Herein, we first introduce the design method and principle of ONNs based on various optical elements. Then, we successively review the non-integrated ONNs consisting of volume optical components and the integrated ONNs composed of on-chip components. Finally, we summarize and discuss the computational density, nonlinearity, scalability, and practical applications of ONNs, and comment on the challenges and perspectives of the ONNs in the future development trends.

## Introduction

Artificial intelligence (AI) has penetrated diverse fields in society and obtained achievements beyond humanity. Neural networks play a crucial role as the core technology supporting the development of AI. In the 1940s, McCulloch and Pitts introduced the working principle of neural networks and the structure of neurons^1,2^. In 1949, Hebb systematically elucidated the theory of neuropsychology^3^. During the 1950s, critical issues regarding the development of AI were discussed and raised^4^, which promoted the crucial technologies in neural networks and the establishment of the discipline of AI. In 1958, Rosenblatt systematically introduced the mathematical model and working principle of perceptron^5^, laying a significant foundation for the further development of neural networks. In 1986, following early pioneering work, Rumelhart, Hinton, and Williams proposed the error back-propagation algorithm to train multi-layer perceptrons^6^. In 1990, LeCun et al. introduced a convolutional neural network based on the back-propagation algorithm and demonstrated its application performance in handwritten digit recognition^7^. In 2012, Krizhevksy et al. proposed deep convolutional neural networks^8^, which further improved the inference abilities of neural networks and made the application fields of AI more extensive^9–13^.

In the meantime, the rapid development of semiconductor process technology has created a host of advanced electronic computing hardware^14–20^ with better performance than CPUs in the past decade. The advanced computing hardware predominantly ensures the computing efficiency requirements of neural networks during the iteration process, thereby promoting the rapid development and application of AI in multitudinous fields. However, the machining accuracy of semiconductor manufacturing technology has approached 3nm^21^, and the size has drawn near the physical limit of the transistor. Transistors with such sizes are highly susceptible to quantum tunneling and thermal effects, making them difficult to work well. Accordingly, improving the machining accuracy of semiconductor processes to acquire higher computing power will be unsustainable. The development of neural network technology relies on massive data, advanced algorithms, high computing power, and contemporary social demands. In the future, neural networks may face bottlenecks in meeting computing power requirements during training or inference processes. Therefore, exploring a new computational paradigm is of great significance.

**Fig. 1:**
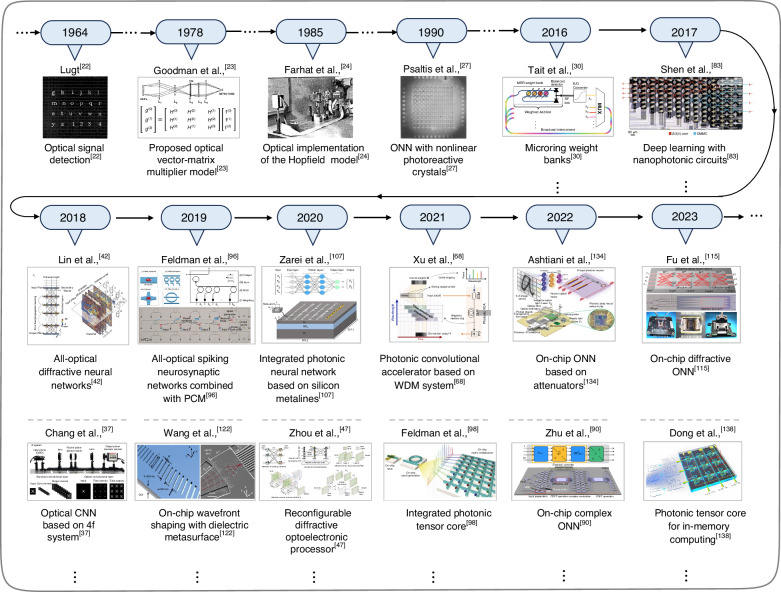
**Timeline of optical neural networks (ONNs) and related optical implementations**. Selected partial key milestones and publications are displayed to retrospect the developments of ONNs. Reprinted from refs. ^22,30^ with the permission of IEEE Publishing. Adapted or reproduced with permission from refs. ^23,24,107^ from © Optical Society of America. Reprinted or reproduced from refs. ^27,68,96,98,134^ with permission from Springer Nature: Nature. Reproduced from refs. ^47,83,138^ with permission from Springer Nature: Nature Photonics. Reprinted by permission from AAAS^42^. Reprinted from ref. ^37^ with permission from Springer Nature: Scientific Reports. Reproduced from refs. ^90,115,122^ with permission from Springer Nature: Nature Communications

Extensive matrix operations in the training or inferring stage of neural networks can be equivalent to the propagation process of light. Due to the advantages of low latency, low power consumption, large bandwidth, and parallel signal processing of light, the matrix operations process can be executed by modulating the optical feature quantities (amplitude, phase, polarization, angular momentum, etc.) during the light propagation process. Thus, further designing optical systems to achieve the inference function of ONNs is more advantageous than its electronic counterparts. As early as the 1960s and 1970s, preliminary basic research regarding ONNs was carried out, e.g., optical signal detection^22^ and optical signal transmission in optical systems^23^. Afterward, in 1985, Farhat et al.^24^ proposed an optical implementation method for the Hopfield model. In 1987, Fisher et al.^25^ presented a method for implementing optical networks with variable adaptive learning capability. In 1989, Caulfield et al.^26^ systematically introduced ONNs and pointed out that ONNs emerged from the mutual infiltration of traditional optical information processing and neural network knowledge systems. Caulfield et al.^26^ consider ONNs would be superior to electronic neural networks in certain situations. In 1990, Psaltis et al.^27^ introduced a method for implementing nonlinear functionality in ONNs using photoreactive crystals. In 1994, Reck et al.^28^ used optical devices such as beam splitters and phase shifters to achieve the operation function of unitary matrices in a cascaded manner. In 2016, Clements et al.^29^ proposed a matrix factorization method based on the Mach–Zehnder interferometer (MZI) cascade approach. Tait et al.^30^ achieved wavelength filtering and power allocation for micro-ring resonators (MRRs). These research works have laid a solid and crucial theoretical foundation for the subsequent development of ONNs. Figure 1 shows the timeline of partial emblematic works in the progression of ONNs.

**Fig. 2:**
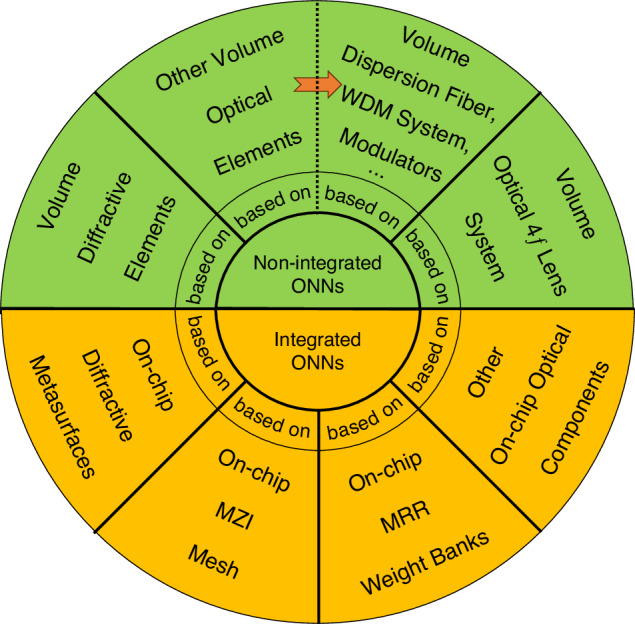
**Classification diagram of optical neural networks**. Non-integrated ONNs consist of volume optical elements; Integrated ONNs consist of on-chip optical components

ONNs have made hosts of research progress^31–35^ through the continuous exploration and efforts of predecessors. This article will provide a comprehensive introduction to the evolution of ONNs in recent years. Firstly, we briefly introduce the principle of ONNs, including the structure of ONNs, the model of optical neurons, and the implementation methods of matrix multiplication function. Secondly, we systematically survey the research of ONNs in recent years from two modules (divided into seven aspects, Fig. 2), including non-integrated ONNs based on volume optical elements and integrated ONNs composed of on-chip optical components. Finally, we summarize and analyze the current advantages and challenges of ONNs regarding computational density, nonlinearity, scalability, practical applications, etc. We will also provide an outlook and discussion on the future development trends of ONNs.

## Principle of ONNs

### Biological neuron model and artificial neural network

**Fig. 3:**
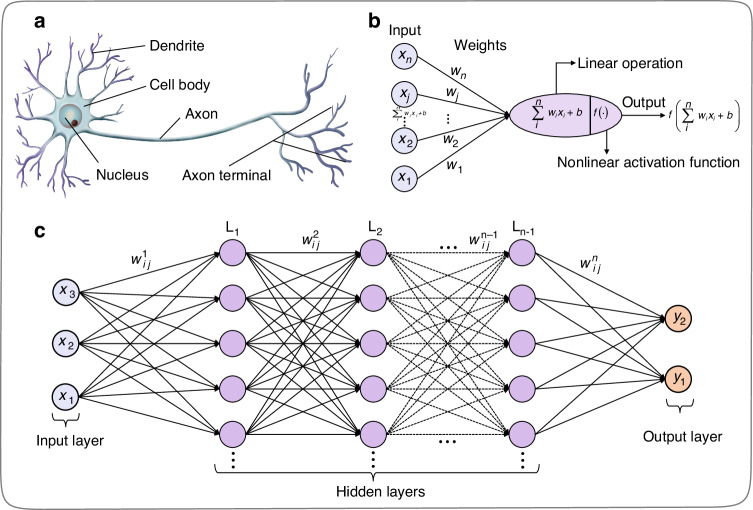
**Neuron structure and artificial neural network. a** Structure of biological neurons. **b** Mathematical inferring process of artificial neurons in multi-layer perceptron, including the input, weights, summation, activation function, and output. **c** Multi-layer perceptron artificial neural network

Neural networks are mathematical models established by imitating the human brain’s nervous system. The functions effectuated by artificial neurons mimic those of dendrites, cell nucleus, axons, and synapses in biological neurons. Figure 3a, b show the abstract structure of a biological neuron and its logical inferring model. In Fig. 3b, ${{\boldsymbol{w}}}_{{\boldsymbol{n}}}$ ($n=\mathrm{1,2},\ldots$) is referred to as a weight, which is a parameter that controls the importance of the input signal. $b$ is bias, which is a parameter that adjusts the ease of activation of neurons. Furthermore, artificial neurons can construct a neural network (multi-layer perceptron) by combining various weight connections, as depicted in Fig. 3c.

### Implementation of optical matrix operations

**Fig. 4:**
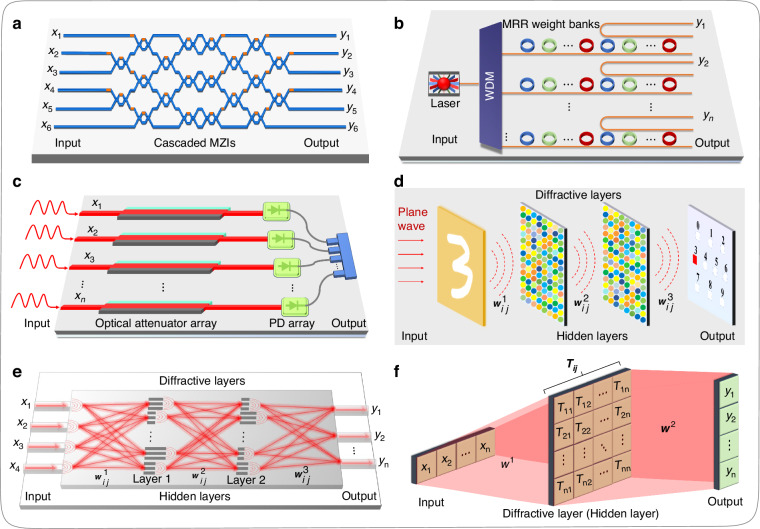
**Different optical elements and systems for implementing optical matrix multiplication. a** Optical matrix multiplication (OMM) implementation based on MZI cascade system. **b** OMM implementation based on wavelength-division multiplexing (WDM) system. **c** OMM implementation based on attenuator array. **d** OMM implementation based on free-space diffractive metasurfaces. **e** OMM implementation based on integrated metalines (consists of subwavelength diffractive units). **f** Mathematical representation visually displays of the physical inferring process of optical neural networks based on 4*f* system or diffractive elements

As shown in Fig. 3c, the neural network consists of many interconnected neurons, and the connections between these neurons are termed as weights. The neurons on the first hidden layer interact with the input signals through the first weight matrix (${{\boldsymbol{w}}}_{{\boldsymbol{ij}}}^{{\bf{1}}}$), and then such neurons transmit the calculated results as a new signal to the neurons on the next hidden layer. Each neuron in the entire neural network repeats the above process until the input signals reach the output end. Optical systems can achieve the function of matrix multiplications, and the implementation methods among different optical systems are diverse, including MZI mesh (Fig. 4a), MRR weight banks (Fig. 4b), and other optical components (Fig. 4c). It is worth noting that the weight matrix values implemented by the optical system mentioned above can correspond one-to-one with the weight matrix values in Fig. 3c, which can be obtained through training by optimization algorithms. However, for ONNs constructed based on diffractive components, namely diffractive optical neural networks (DONNs), which are shown in Figs. 4d, e, the weights (${{\boldsymbol{w}}}_{{\boldsymbol{ij}}}^{{\boldsymbol{n}}},n=1,2,\ldots$) between the adjacent layers, such as the input layer, hidden layers, and the output layer, are fixed due to the connection of neurons through light diffraction. The training parameters of hidden layers (such as ${{\boldsymbol{T}}}_{{\boldsymbol{ij}}}$ in Fig. 4f) are usually the transmission coefficients of subwavelength structures on the diffractive layers. The weight matrix (${{\boldsymbol{T}}}_{{\boldsymbol{ij}}}$) that DONN needs to train is different from the weight matrix values (${{\boldsymbol{w}}}_{{\boldsymbol{ij}}}^{{\boldsymbol{n}}}$) of the ONNs based on the MZI mesh, MRR weight banks, and other optical components.

## Non-integrated ONNs based on volume elements

### ONNs based on volume optical 4*f* system

The optical 4*f* system is a typical optical transfer system consisting of two lenses with a focal length of *f*, which can achieve a Fourier transform of the input optical field. After the input signal is transformed from the time domain to the frequency domain, the frequency spectrum of the input signal can be modified or modulated conveniently in the Fourier plane to obtain the corresponding output response. The work of using a 4*f* system to perform matrix multiplication has been validated^36^. The frequency domain information at the Fourier plane can be designed and modulated to achieve the inference function of ONNs^37–41^.

**Fig. 5:**
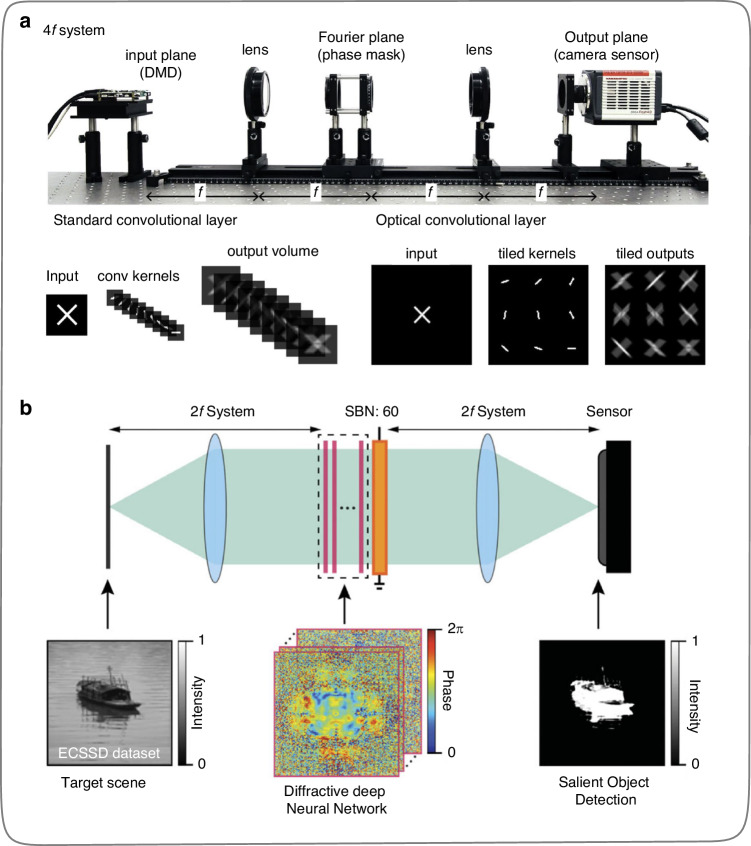
**ONNs implementation based on 4*f*** **system. a** Optoelectronic hybrid convolutional neural network with phase mask placed in the Fourier plane of the 4*f* system^37^. **b** All-optical neural network with diffractive depth neural network placed in the Fourier plane of the 4*f* system^38^. **a** Reprinted from ref. ^37^ with permission from Springer Nature: Scientific Reports. **b** Reprinted with permission from ref. ^38^ from © The American Physical Society

In 2018, Chang et al.^37^ proposed a scheme for devising optical convolutional neural networks (OCNNs) based on the 4*f* system (Fig. 5a), which involves placing a phase mask in the Fourier plane of the 4*f* system to achieve the function of convolutional kernels. Although the convolutional kernels cannot support parameter reconfiguration, the OCNN brings lower training costs, lower computation, and better predictive performance to the optoelectronic hybrid systems. In this work, the nonlinear function is fulfilled in the electrical domain. In July 2019, Yan et al.^38^ placed a diffractive depth neural network (D^2^NN) in the Fourier plane of a 4*f* system (Fig. 5b) through numerical simulation, which significantly improved the classification accuracy and robustness of the optical system by adding an optical nonlinear activation function (optical characteristic parameters of ferroelectric thin films) to the D^2^NN. Yan et al.^38^ compared and analyzed the performance of the optical system after training with/without nonlinear activation layers of ferroelectric thin films introduced during the training process, as well as the number of nonlinear activation layers introduced. This work may provide theoretical guidance for designing and fabricating nonlinear devices in the subsequent 4*f* system.

**Fig. 6:**
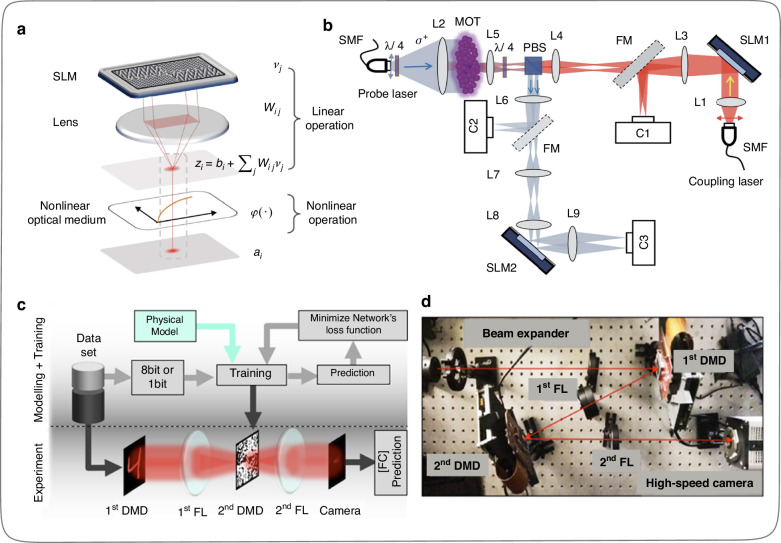
**Reconfigurable ONNs implementation based on 4*f*** **system. a** Calculation process implementation of a single neuron, including linear and nonlinear operation^39^. **b** Reconfigurable ONNs built on 4*f* system and SLMs^39^, the nonlinear optical activation function in the optical system is realized based on electromagnetically induced transparency^161,162^. **c** Reconfigurable ONNs built on 4 *f* system and DMDs^40^. **d** Implementation of experimental setup^40^ for (**c**). **a**, **b** Reprinted with permission from ref. ^39^ from © Optical Society of America. **c**, **d** Reprinted with permission from ref. ^40^ from © Optical Society of America

In August 2019, Zou et al.^39^ used a spatial light modulator (SLM) and Fourier lens to complete the input signal loading and weight matrix construction, and the introduced SLM made the matrix operation process programmable in real-time. In addition, Zou et al.^39^ introduced nonlinear optical media in the matrix operation process, endowing the operation process of ONNs with nonlinearity. The specific physical implementation method is shown in Fig. 6a. On this basis, the research group used two SLMs and a 4*f* system group to complete a two-layer all-optical neural network with nonlinear and reconfigurable functions, as shown in Fig. 6b. This work enhances the functionality (implemented nonlinearity and reconfigurability) of the ONN based on the 4*f* system and endows it with more powerful logical inferring abilities. In 2020, Miscuglio et al.^40^ proposed a massively parallel amplitude-only Fourier ONN (Figs. 6c, d). Specifically, the kilohertz-fast reprogrammable high-resolution digital micromirror devices (DMDs) were introduced in the 4*f* system to implement input signal loading and Fourier plane signal modulation. The modulation rate of DMD is at least 2 orders of magnitude faster than that of SLM with the same pixel resolution, making the ONN system faster and more efficient.

### ONNs based on discrete optical diffractive elements

**Fig. 7:**
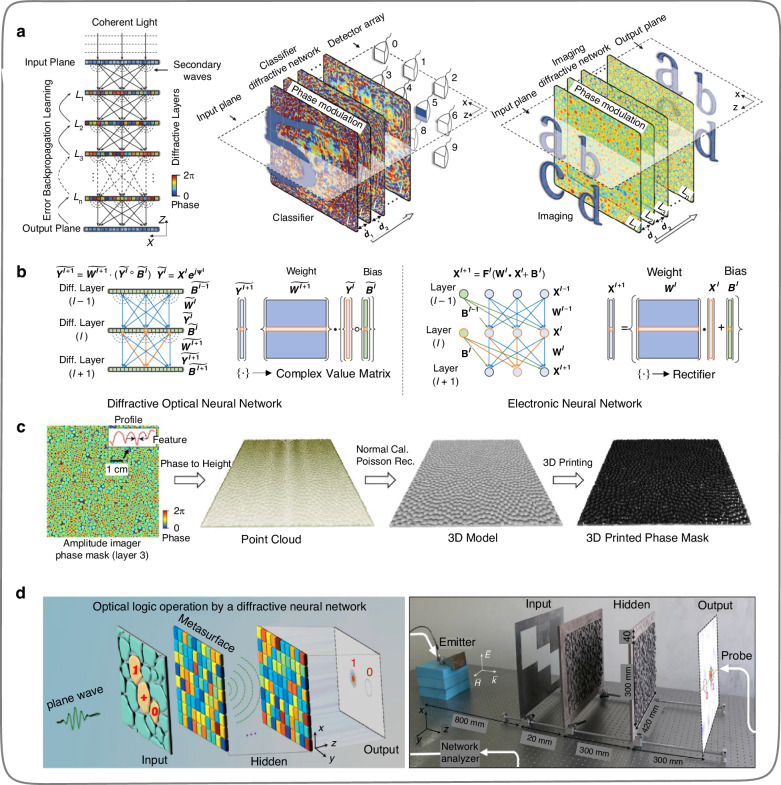
**Diffractive deep neural network (D**^**2**^**NN). a** Schematic diagram of the physical inferring process of D^2^NN^42^. **b** Comparison between a D^2^NN and an electronic conventional neural network^42^. **c** 3D model reconstruction of a D^2^NN hidden layer for 3D-printing^42^. **d** Schematic illustration and experiment setup of optical logic operations by a DONN^44^. **a**-**c** Reproduced by permission from AAAS^42^. **d** Reproduced from ref. ^44^ with permission of Springer Nature: Light: Science & Applications

The trainable parameters (modulation units) of the ONNs built based on the 4*f* system are used to be placed in the Fourier plane^37–40^, which limits the expansion of trainable parameters and the number of hidden layers. However, free-space DONNs constructed by diffractive elements^42–59^ can overcome these limitations well. In 2018, Lin et al.^42^ proposed an all-optical deep learning framework, in which neural networks are physically formed by multiple layers of diffractive surfaces (Fig. 7a). The diffractive surfaces are composed of diffractive units, each of which is termed as a hidden layer in DONNs, and each diffractive unit on the diffractive surface is defined as a neuron. Meanwhile, the function of the diffractive unit is to change the phase difference after the light passes, and the specific phase difference values can be pre-trained on the computer through intelligent algorithms such as forward propagation, gradient descent, error back-propagation, etc. Among them, the forward propagation process (wave analysis) is demonstrated by the Rayleigh-Sommerfeld diffraction equation^42^. After obtaining all phase difference values, the diffractive surfaces can be fabricated using 3D printing technology to obtain the physical implementation device of DONNs (Fig. 7c). The relationship between the thickness of diffractive units and phase difference values is linear, and the specific value of the thickness of each diffractive unit can be calculated by the formula: $h=\lambda \phi /\left(2\pi \varDelta n\right)$, where ∆*n* is the refractive index difference between the 3D printing material and air, $\phi$ is the phase difference value (trainable parameter), $\lambda$ is the wavelength of light propagation in free-space. Finally, Lin et al.^42^ validated the classification tasks of the MNIST and Fashion MNIST datasets by encoding the input signal into the amplitude and phase of light, respectively. This work proposed a novel scheme for the study of ONNs. In 2 years, Qian et al.^44^ designed a DONN (Fig. 7d) for logic gate operations (AND, OR, NOT, etc.) based on Rayleigh-Sommerfeld diffraction. The hidden layers function of the DONN is implemented by the Huygens metasurfaces fabricated through mechanical processing.

**Fig. 8:**
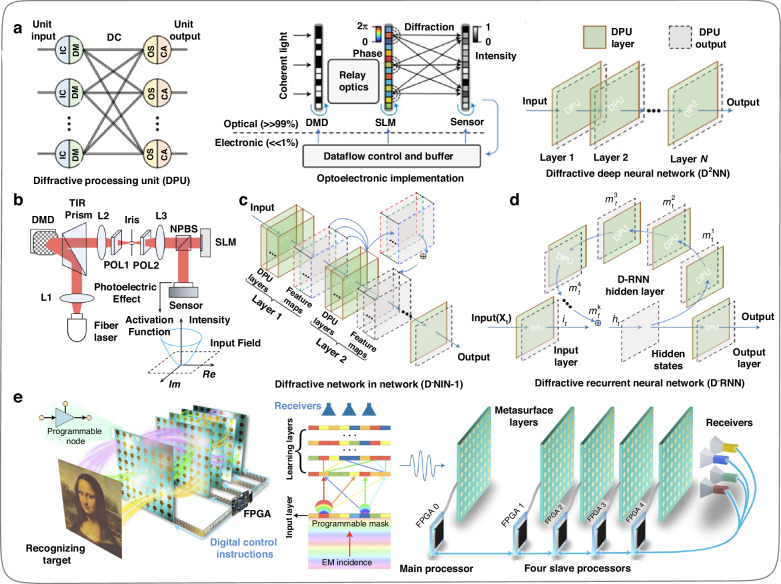
**Progress and expansion of the diffractive optical neural network (DONN). a** Reconfigurable diffractive processor unit (DPU)^47^. **b** Schematic of the DPU prototype^47^. **c** Feedforward DONN built based on DPU^47^. **d** Recurrent DONN built based on DPU^47^. **e** Programmable DONN based on digital-coding metasurfaces^53^. **a**–**d** Reproduced from ref. ^47^ with permission of Springer Nature: Nature Photonics. **e** Reproduced from ref. ^53^ with permission of Springer Nature: Nature Electronics

Noteworthiness, the current DONNs^42,44^ does not have reconfigurable functionality, and nonlinearity is not introduced in the network except for the output layer (optical intensity detection^60^). In addition, the fabricating errors generated during the machining process might accumulate with the depth increases of ONNs, resulting in inevitable systematic errors. The difficulties mentioned above extensively limit the model complexity and experimental performance of existing DONN processors. Thus, in 2021, Zhou et al.^47^ proposed a reconfigurable diffractive processing unit (DPU), as shown in Fig. 8a, based on which an optoelectronic fusion computing architecture can be constructed, and this architecture can support different neural networks and achieve high model complexity with millions of neurons. The DPU consists of an input layer, an information processing layer, and an output layer. The input data of the DPU input layer is optically encoded by DMD, and the physical process of the information processing layer is implemented by SLM and optical diffraction. The optical field summation and nonlinear activation function of the output layer are achieved by the photoelectric effect on each pixel of the complementary metal-oxide–semiconductor (CMOS) sensor (Fig. 8b). In this work, Zhou et al.^47^ designed a feedforward deep ONN (Fig. 8c) and a diffractive recurrent ONN (Fig. 8d) using DPU as the basic units. In 2022, Liu et al.^53^ designed a programmable DONN (Fig. 8e) based on information metasurfaces^61^. The meta-structure (neuron) arrays on such a metasurface (hidden layer) can be uniformly routed and controlled through the field-programmable gate arrays (FPGA), thereby achieving real-time control of the amplitude and phase coefficients of each neuron. In other words, the values of each neuron on the DONN hidden layers can be flexibly set. This work is expected to promote the application of DONN in the microwave fields, such as remote control, wireless communication, signal enhancement, medical imaging, and the Internet of Things. It is worth mentioning that the introduction of programmable information metasurfaces enhances the programmability of DONNs, and embedding optical power amplification devices on each diffractive unit is conducive to further expanding the depth of DONNs.

### ONNs based on other volume optical components

**Fig. 9:**
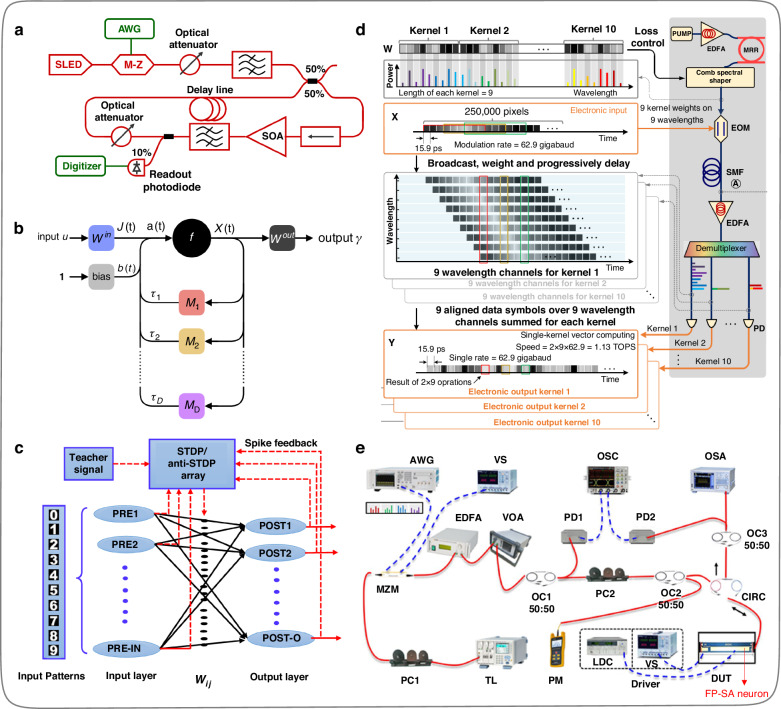
**ONNs constructed by various volume optical components. a** Schematic of the experimental set-up of the all-optical reservoir^62^. **b** Scheme of the Folded-in-time deep neural network^71^. **c** Architecture of the VCSEL-based all-optical spiking neural network^69^. **d** Optical convolution accelerator designed by time-wavelength interleaving multiplexing technique^68^. **e** Experimental setup to test the FP-SA neuron^73^. **a** Reprinted with permission from ref. ^62^ from © Optical Society of America. **b** Reproduced from ref. ^71^ with permission of Springer Nature: Nature Communications. **c** Reprinted from ref. ^69^ with the permission of IEEE Publishing. **d** Reproduced from ref. ^68^ with permission of Springer Nature: Nature. **e** Reprinted with permission from ref. ^73^ from © Optical Society of America

Other volume optical components refer to the ordinary single-mode fibers, dispersion fibers, modulators, attenuators, filters, Fabry-Perot laser with saturable absorber, polarizing beam splitter, wavelength-division multiplexing (WDM) system, etc., which can flexibly construct ONNs in different combinations^62–76^. In 2012, Duport et al.^62^ achieved the experimental demonstration of all-optical reservoir calculation for the first time based on single-mode fibers, semiconductor optical amplifiers (SOA), tunable optical attenuators, delay loops, bandpass filters, etc. (Fig. 9a). Meanwhile, the nonlinearity is provided by the saturation of the optical gain in the SOA. In 2021, Stelzer et al.^71^ constructed the computational function of a single neuron using a nonlinear device and multiple time-delay feedback loops and implemented a deep neural network of any size by continuously iteratively reconstructing the parameters of the single neuron. The network’s connection weights were implemented by adjusting the feedback modulation signal and delay within the loop, as shown in Fig. 9b.

In 2021, Xiang et al.^69^ designed photonic neurons and synapses based on the vertical-cavity surface-emitting laser with an embedding saturable absorber (VCSEL-SA) and the vertical-cavity semiconductor optical amplifiers (VCSOA) respectively, and based on which they constructed an all-optical spiking neural network (SNN), as shown in Fig. 9c. This work built up a new framework for classification tasks through supervised learning manner and developed a self-consistent unified neuron-synapse-learning model, providing a hardware-friendly method for implementing SNN in the optical domain. In the same year, Xu et al.^68^ designed a universal optical vector convolution accelerator adopting the time-wavelength interleaving method (Fig. 9d), and then implemented the function of the optical convolution accelerator on hardware based on an optical frequency comb and WDM system, reaching a computing speed at more than 10 TOPS (trillions of operations per second). The input of the optical convolution accelerator is completed through an on-chip optical frequency comb, while its output, weight matrix operation process, and convolution kernel allocation are completed through a WDM system. Furthermore, Xu et al.^68^ designed an optical convolutional neural network for recognizing handwritten digital images, with an experimental recognition accuracy of 88%, which is very close to 90% of the numerical calculation results obtained through optimization training. The method proposed by Xu et al.^68^ can be used for training more complex networks, which is promising to be applied to complex scenes such as autonomous vehicles and real-time video recognition. In 2023, Xiang et al.^73^ developed a photonic spiking neuron chip based on an integrated Fabry-Perot laser with a saturable absorber (FP-SA), which provides an indispensable foundational module for constructing photonic spiking neural network (PSNN) hardware (Fig. 9e). Additionally, they proposed time-multiplexed temporal spike encoding to achieve a functional PSNN that far exceeds hardware integration scale limitations, paving the way for multi-layer PSNN with nonlinearity that can handle complex tasks. Besides designing spiking neurons based on FP-SA, many other methods can also achieve the functionality of spiking neurons^77–81^, and further construct PSNNs.

### Integrated ONNs based on on-chip optical components

With the assistance of advanced semiconductor process technologies, miniaturization proves to be a significant trend in the development of ONNs. Compared with ONNs constructed with volume optical elements, on-chip integrated ONNs have advantages such as high computational density, portability, and stability, which may play a crucial role in the birth of new computing machines (e.g., optical computers). This section will introduce the research on integrated ONNs based on various on-chip optical components.

### ONNs based on on-chip MZI mesh

The propagation of light is a natural process of matrix operation. In 1994, Reck et al.^28^ conducted experimental verification of unitary matrix operation using traditional bulk optical components (beam splitters and phase shifters). On this basis, Clements et al.^29^ proposed an optimized matrix factorization method in 2016, which makes optical components more efficient in achieving matrix factorization. The same year, Ribeiro et al.^82^ implemented a reconfigurable 4$\times$4-dimensional matrix by cascading on-chip MZIs. From then on, many on-chip ONNs^60,83–92^ based on MZI mesh have emerged.

**Fig. 10:**
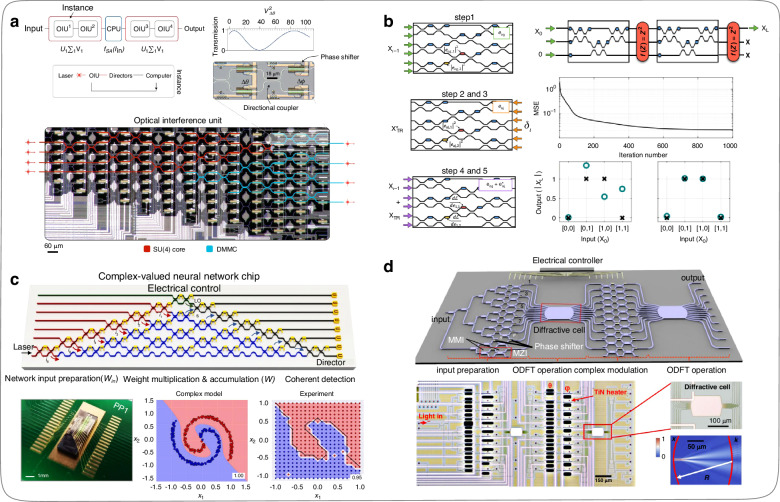
**ONNs constructed by MZIs. a** On-chip ONN based on 56 MZIs^83^. **b** Mathematical inference diagram of the training process for ONN that supports in-situ online training^84^. **c** On-chip ONNs based on amplitude and phase modulation^60^. **d** ONN chip designed and fabricated based on MZIs and diffractive units^90^. **a** Reproduced from ref. ^83^ with permission of Springer Nature: Nature Photonics. **b** Reproduced with permission from ref. ^84^ from © Optical Society of America. **c** Reproduced from ref. ^60^ with permission of Springer Nature: Nature Communications. **d** Reproduced from ref. ^90^ with permission of Springer Nature: Nature Communications

In 2017, Shen et al.^83^ designed an ONN with a matrix dimension of 4$\times$4 by cascading 56 MZIs and completed the fabrication of an ONN chip on a silicon-based substrate, as shown in Fig. 10a. Theoretically, any matrix can be decomposed into one diagonal matrix and two unitary matrices by using the singular value decomposition method. The optical attenuators can implement any diagonal matrix function, and the beam splitters and phase shifters can achieve any unitary matrix function. Thus, the training weight matrices of ONNs can be physically implemented one-to-one via integrated optical elements. In this work, the parameters of on-chip ONN are obtained through pre-training on a computer, and the optical transmission characteristic curve of a saturated absorber is adopted as the nonlinear function during the ONN training process. Afterward, Shen et al.^83^ completed the 4$\times$4-dimension matrix operation by reconstructing on-chip MZIs step by step in the ONN inferring stage, which achieved a blind test accuracy of 76.7% in the vowel recognition dataset in experiments. This work leads to a new method for studying on-chip ONNs. In 2018, Hughes et al.^84^ proposed an in-situ training method for on-chip ONNs designed by MZI mesh. Firstly, the optical power of the output port of the on-chip ONNs is accurately measured. Then, the adjoint variable method is employed to realize the gradient derivation and error back-propagation during the on-chip ONNs in-situ training process (Fig. 10b). This method allows the structural parameters of on-chip ONNs to be directly trained by optical hardware, thus overcoming the error problem caused by the chip fabricating process, which is a crucial advancement in the training method of on-chip ONNs based on MZI mesh, from offline training to in-situ online training. In addition, this work also has a reference for the research of optoelectronic fusion ONNs, such as how to solve the problems in the process of repeated signal conversion between optical chips and electronic hardware.

In 2021, Zhang et al.^60^ pointed out that most research on ONNs still only uses traditional real-value frameworks designed for digital computers. Therefore, the team utilized both the phase and amplitude of light during the training process of ONNs, resulting in higher degrees of freedom for the optimized structural parameters of ONNs during the training process (doubling the adjustable variables). Consequently, the trained ONNs (Fig. 10c) exhibit superior logical inferring abilities. The ONNs have completed tasks such as logic gate operation, IRIS dataset category prediction, nonlinear data (circle and spiral) classification, and MNIST dataset handwritten digit recognition. The performance of ONNs obtained by this design method is better, with classification accuracy and convergence speed of the loss function compared to other ONNs designed based on MZI mesh at the same matrix size, prominently improving the computational speed and energy efficiency of ONNs. The following year, Zhu et al.^78^, who belonged to the same research group as Zhang et al.^60^, went further in their existing study by introducing integrated diffractive elements that can implement the Fourier transform and inverse transform (Fig. 10d), thereby improving the matrix dimensions resolved by ONNs during the operation process and reducing their computational energy consumption. Compared with the previous work^60^, this new design scheme outperformed ONNs solely based on MZIs topology cascading regarding integration and energy consumption in classification experiments on the same IRIS and MNIST datasets.

### ONN based on on-chip MRR weight banks

**Fig. 11:**
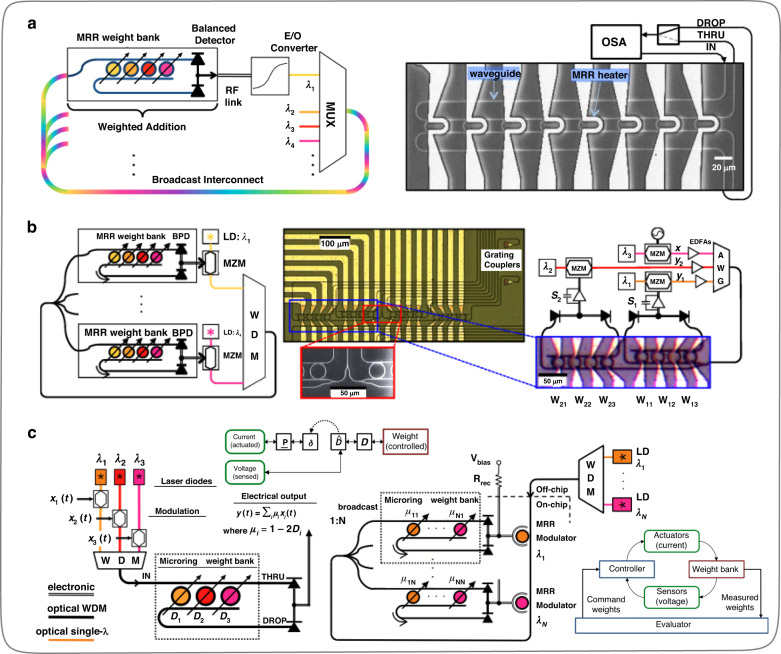
**The implementation foundation of ONNs, MRR weight banks. a** Weights configuration verification of MRRs^30^. **b** Implement ONNs using MRR weight banks^93^. **c** Feedback control for MRR weight banks^94^. **a** Reproduced from ref. ^30^ with the permission of IEEE Publishing. **b** Reproduced from ref. ^93^ with permission of Springer Nature: Scientific Reports. **c** Reproduced from ref. ^94^ with the permission of IEEE Publishing

MRR has a filtering function and can regulate the optical power of different wavelengths. In 2016, Tait et al.^30^ conducted a detailed study on the MRR weight banks (Fig. 11a), including the principle of MRR, mutual channel crosstalk, and its design methods. They predict that the MRR weight banks may unlock brand new domains of computing based on silicon photonics. After, in 2017, Tait et al.^93^ used the MRR weight banks to configure the connection weights of ONNs (Fig. 11b), proving the mathematical isomorphism between silicon photonic circuits and continuous neural network models. In addition, the team derived and analyzed the power consumption of modulator-class neurons. Further, in 2018, Tait et al.^94^ investigated the fabrication process and thermal sensitivity challenges of MRRs, rendering a feedback weight control method (Fig. 11c) to overcome the weight control problem in reconfigurable photonic networks. Research labors by Tait et al.^30,93,94^ have laid a significant foundation for the subsequent developments of ONNs based on MRR weight banks.

**Fig. 12:**
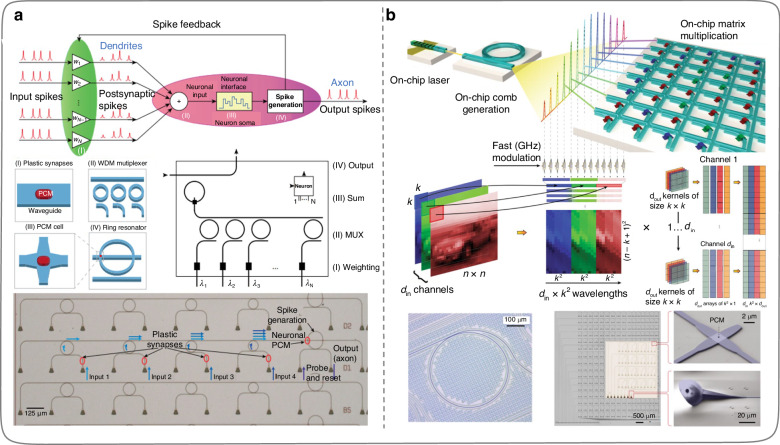
**ONNs implementation by MRRs and PCM units. a** Principle and experimental diagram of the all-optical spiking neurosynaptic networks^96^. **b** Photonic in-memory computing using a photonic-chip-based microcomb and PCM units^98^. **a** Reproduced from ref. ^96^ with permission of Springer Nature: Nature. **b** Reproduced from ref. ^98^ with permission of Springer Nature: Nature

Following the research of predecessors, new research on ONNs^95–105^ based on MRR weight banks has emerged one after another. In 2019, Feldmann et al.^96^ designed a spiking all-optical synaptic system (Fig. 12a) based on MRRs and phase change material (PCM), which avoids the physical separation of memory and processor in conventional computing architectures. Therefore, the inference process of the spiking ONN designed by Feldmann et al.^96^ is more analogous to the brain. The specific working principle of the ONNs implemented by this method is to use PCM units to weight the input pulses, and then couple the corresponding wavelength of light into a single-mode waveguide through MRRs for power summation. When the accumulated optical power in the single-mode waveguide exceeds a certain threshold, the PCM unit on the last MRR will switch the crystal state and generate an output pulse, completing a calculation on the optical domain. Meanwhile, the optical nonlinearity in the inferring process of the ONNs is physically implemented through the PCM unit on the last MRR. Two years later, Feldmann et al.^98^ further designed and fabricated an integrated photonic processor (Fig. 12b) on the silicon nitride platform, which parallelly implemented the processing function of traditional convolutional kernels in an all-optical manner on the chip. The operating speed of the photonic processor can reach trillions of operations per second. PCM has the characteristic of nonvolatility. The nonlinearity and reconfigurability of the on-chip ONNs can be realized by introducing PCM units on integrated chips. Feldmann et al.^96,98^ provide a valuable reference for the subsequent developments of all-optical ONNs.

**Fig. 13:**
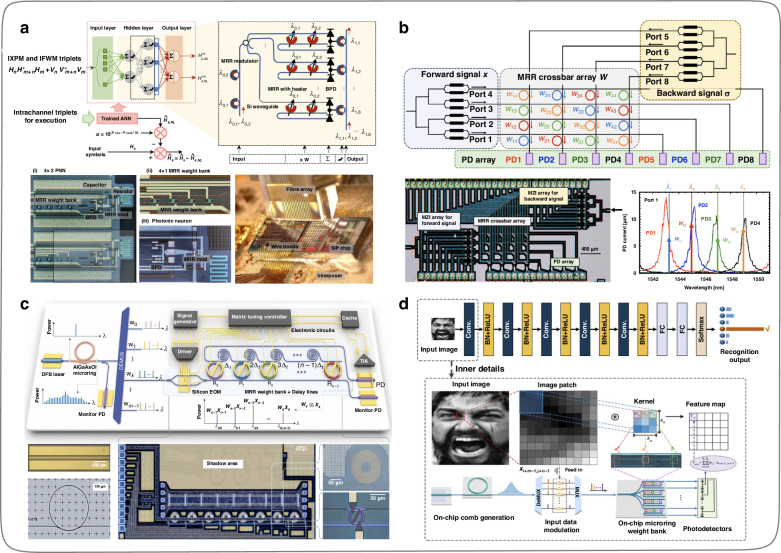
**ONNs implementation by MRR weight banks. a** ONN for nonlinear compensation in long-distance fiber optic links^99^. **b** The photonic integrated circuit system designed based on the crossbar framework, which supports training the structural parameters of ONNs online^100^. **c** ONN system with the light source, data loading area, and data processing units on a single chip^103^. **d** Convolution operations for human emotion recognition by a microcomb-enabled integrated ONN^105^. **a** Reproduced from ref. ^99^ with permission of Springer Nature: Nature Electronics. **b** Reprinted from ref. ^100^ with the permission of ACS Publishing. **c** Reproduced from ref. ^103^ with permission of Springer Nature: Nature Communications. **d** Reproduced from ref. ^105^ with permission of De Gruyter Publishing

In 2021, Huang et al.^99^ designed and fabricated an on-chip ONN based on the MRR weight banks on the silicon-on-insulator (SOI) platform (Fig. 13a). The function of this ONN is to assist electronic hardware systems in completing nonlinear compensation in submarine fiber optic links. As the ONN system can process optical signals in the analog domain, it enormously reduces the high complexity and high-speed requirements of conventional digital signal processing circuits in handling nonlinear compensation in submarine long-distance fiber optic communication links. In 2022, Ohno et al.^100^ pointed out that most of the Si programmable photonic integrated circuits (PICs) proposed for ONNs suffer the issues of low scalability and incomplete all-optical training frameworks. Therefore, Ohno et al.^100^ proposed a crossbar array framework based on the MRR weight banks and designed a PIC based on this framework for parameters online training of on-chip ONNs. The programmable PIC (Fig. 13b) does without complex algorithms such as singular value decomposition during matrix-vector multiplication, supporting a gradient back-propagation algorithm for online training of on-chip ONNs, which is beneficial for integrated system error calibration. In January 2023, Bai et al.^103^ designed an on-chip ONN system (Fig. 13c) using integrated optical frequency combs, MRR weight banks, and silicon-based spiral waveguide delay lines. The multiple wavelength sources, data loading areas, and data processing centers are fully integrated into a single chip. The convolution function is realized by adopting the time-wavelength stretching method, and the computational density can reach about 1.04 TOPS per square millimeter. In October 2023, Cheng et al.^105^ designed a microcomb-enabled integrated ONN based on microcomb and MRR weight banks (Fig. 13d). It can perform tensor convolution operations and complete intelligent tasks for human emotion recognition (6 types of human emotions) at low power consumption and light speed, with a blind testing accuracy of 78.5%. This work is a meaningful attempt by ONNs in practical applications, with a potential throughput of up to 51.2 TOPS, providing a reference for the next generation of computing hardware in intensively computational artificial intelligence applications.

### ONN based on on-chip diffractive metasurfaces

The clever design of metasurfaces can theoretically achieve arbitrary control of the wavefront of reflected/refracted beams^106^. Based on this, research on integrated DONNs^107–120^ has also been carried out.

**Fig. 14:**
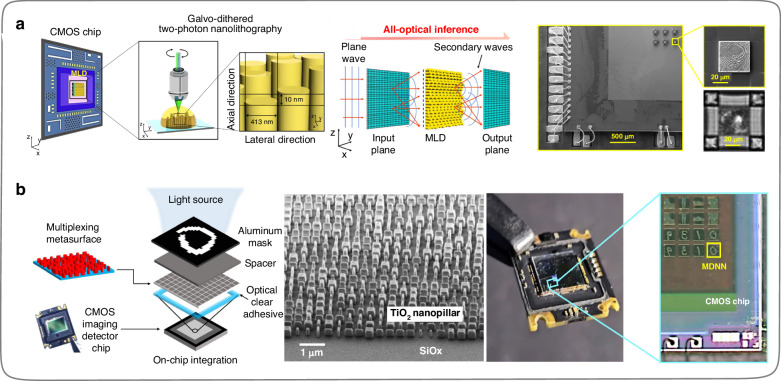
**Diffractive optical neural network (DONN) designed based on metasurface with a CMOS chip substrate. a** High neuron density DONN manufactured based on two-photon nanolithography technology^109^. **b** Polarization multiplexing DONN based on metasurface design^112^. **a** Reproduced from ref. ^109^ with permission of Springer Nature: Light: Science & Applications. **b** Reproduced from ref. ^112^ with permission of Springer Nature: Light: Science & Applications

To reduce the size of computing units and further improve the integration and system stability of the free-space DONNs. In 2021, Goi et al.^109^ selected a near-infrared wavelength (785 nm) and fabricated an integrated DONN with high neuron density by two-photon nanolithography using the complementary metal-oxide semiconductor (COMS) chip as the substrate (Fig. 14a). The research team fabricated a multilayer diffractive metasurface on a CMOS chip consisting of an array of subwavelength cylindrical structures in steps of 10 nm in the height direction of the cylinder. This DONN is highly integrated and can fabricate about 500 million neurons per square centimeter. In 2022, Luo et al.^112^ selected the visible wavelength (532 nm) to fabricate an integrated DONN on a CMOS chip substrate that can support performing multi-channel sensing and multitasks in a visible light environment (Fig. 14b). The metasurface (hidden layer) in the integrated DONN consists of a subwavelength nanopillar array with a fixed height of 600 nm and a central spacing between adjacent nanopillars (the nanopillar array period) of 400 nm, which modulates the phase of propagated light by varying the length and width of each nanopillar. In this work, a multi-channel classifier framework was constructed by implementing a polarization multiplexing scheme using subwavelength nanostructures, which further demonstrates that the comprehensive design of optical feature quantities can endow ONNs architectures with the ability to handle multiple tasks (the various classification tasks of MNIST and Fashion-MNIST were demonstrated). In addition, the number of neurons on this CMOS chip that could be integrated per square millimeter was about 6.25 × 10^16^.

**Fig. 15:**
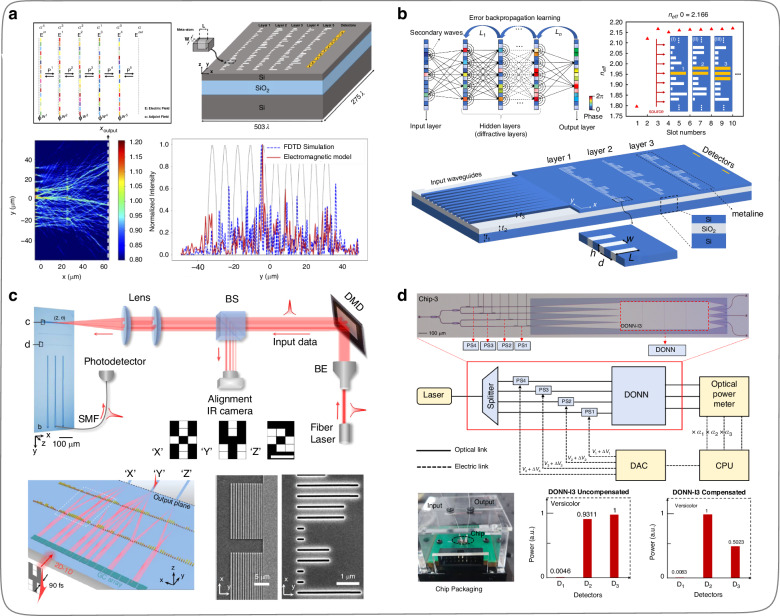
**DONNs implementation by on-chip diffractive metalines. a** Simulation verification of on-chip DONN^107^. **b** Simulation structure and design model of on-chip DONN^108^. **c** On-chip DONN experimental verification and input signal system diagram^110^. **d** On-chip DONN experimental testing set-up and error compensation system diagram^115^. **a** Reproduced with the permission from ref. ^107^ from © Optical Society of America. **b** Reproduced with the permission from ref. ^108^ from © Optical Society of America. **c** Reproduced from ref. ^110^ with permission of Springer Nature: Nature Communications. **d** Reproduced from ref. ^115^ with permission of Springer Nature: Nature Communications

Additionally, the on-chip one-dimensional metasurfaces (metalines) composed of subwavelength diffractive units can also achieve the wavefront shaping of light in slab waveguides^121,122^. The on-chip DONNs constructed from diffractive metalines on slab waveguides have better stability, portability, and scalability. In 2020, Zarei et al.^107^ designed an on-chip DONN composed of diffractive metalines by using subwavelength rectangular slots on the SOI platform and validated the performance of the DONNs (Fig. 15a) through simulation calculations. The computing speed of such an on-chip DONN is about 1.2 × 10^16^ multiply-accumulate operations per second. In 2021, Fu et al.^108^ developed an on-chip integrated two-dimensional spatial electromagnetic propagation model and a weight mapping model, which can complete parameter training via computers for on-chip DONN and ensure the pre-trained parameters accurately map onto the physical devices (Fig. 15b). Two years later, Fu et al.^115^ fabricated on-chip DONNs (Fig. 15d) based on the previous theoretical exploration and considered the practical issues that were difficult to care for during the simulation part. Meanwhile, to effectively reduce the system errors caused by chip fabricating and packaging processes, an in-situ training scheme based on the particle swarm algorithm for error compensation was proposed as well. The effectiveness of this scheme remarkably ensured the performance of the DONN chips and improved the robustness of the experimental testing system. In 2022, Wang et al.^110^ further advanced their previous work^122^ by designing and fabricating an on-chip DONN (Fig. 15c) based on subwavelength diffractive units. In this work, to reduce the mutual interference between adjacent subwavelength diffractive units, Wang et al.^110^ combined two identical subwavelength diffractive units as a new computing cell in the hidden layer of the on-chip DONNs. In addition, the input signals are loaded onto the DONN chip through a DMD and lens system, which indicates that the input dimension of the on-chip DONN can be unrestricted by the number of waveguides, providing a feasible solution for the problem of limited input dimension of on-chip ONNs.

### ONN based on other on-chip optical components

In addition to the preceding introduced integrated optical devices^83,90,96,103,109,115^, there are a variety of on-chip optical components that can construct ONNs^123–140^, such as single-mode waveguides, multi-mode interferometers, phase shifters, attenuators, detectors, three-dimensional integrated waveguides, etc.

**Fig. 16:**
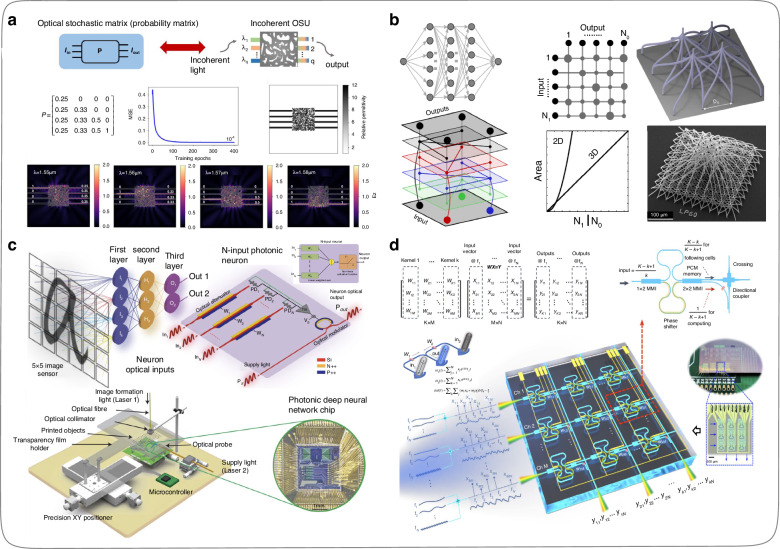
**Various integrated optical components for constructing on-chip ONNs. a** Incoherent optical scattering unit and optical stochastic matrix^129^. **b** Three-dimensional interconnect waveguides of constructing ONNs^128^. **c** Architecture and implementation diagram of the ONN chip^134^. **d** Data architecture and working principle of a photonic tensor core^138^. **a** Reproduced from ref. ^129^ with the permission of Science China Press Publishing. **b** Reproduced with the permission from ref. ^128^ from © Optical Society of America. **c** Reproduced from ref. ^134^ with permission of Springer Nature: Nature. **d** Reproduced from ref. ^138^ with permission of Springer Nature: Nature Photonics

In April 2020, Qu et al.^129^ proposed an optical stochastic architecture based on optical scattering units (Fig. 16a). They adopt the inverse design method to optimize the parameters of the optical scattering units, thereby obtaining on-chip ONNs for deep learning tasks with fast speed, low power consumption, and small footprint. The team designed an on-chip ONN for the MNIST dataset and achieved a prediction accuracy of 97.1% on the blind test set. Theoretically, the inverse design approach can implement any function of the optical scattering units. The advantage of inverse design is that it can achieve target optimization results without knowing the analytical process of parameter acquisition. However, this method has a fatal limitation, such as the optimization process of parameters is time-consuming and demands massive computing power when solving complex tasks. In June 2020, Moughames et al.^128^ utilized two-photon polymer printing technology to fabricate on-chip three-dimensional integrated low-loss photonic waveguide arrays. The waveguides interconnect structure corresponds to large-scale vector-matrix products (Fig. 16b) and is the core of neural network computing. In this work, the diameter of the photonic waveguide is 1.2 μm and the spacing between adjacent waveguides is 20 μm. Consequently, there is about 2200 neurons can be integrated per cubic millimeter through the two-photon polymer printing technology. This scheme provides a novel direction for the development of on-chip ONNs.

In 2022, Ashtiani et al.^134^ proposed and fabricated a silicon-based integrated system using on-chip photonic devices such as attenuators and detectors, which can perform the inference function of ONNs in steps (Fig. 16c). Specifically, the integrated system can only perform one calculation process for a single neuron at one time, which demands modulating the coefficients of the attenuators multiple times to complete weight allocation and matrix operation. Although the computing capacity of the on-chip ONNs is low, it has advantages such as simple structure, reconfigurability, and nonlinearity. In 2023, Dong et al.^138^ proposed a method for three-dimensional data processing, which introduced radio-frequency modulation of photonic signals to increase parallelism, thereby adding the input dimensions of the on-chip ONNs based on spatially distributed nonvolatile memory and wavelength multiplexing. The optical system constructed by the photonic tensor core (Fig. 16d) attaches parallelism of 100 degrees, achieving two orders of magnitude higher than using only spatial and wavelength interleaving methods. This work provides a significant inspiration for solving the input dimension limitation problem of on-chip ONNs. Subsequent research regarding on-chip ONNs can consider simultaneously mapping data features onto multiple feature quantities of light, thereby increasing the dimensionality of input data in the limited physical input channels.

## Discussion

ONNs have been developing for decades since the 1960s. This review summarizes seven different optical devices designed for ONNs under the two major modules of non-integrated ONNs and integrated ONNs. In this chapter, we score these ONNs regarding integration level, computing capacity, stability/portability, universality, reconfigurability, nonlinearity, and scalability. Then, we analyze and summarize the corresponding performances of the various ONNs and envisage their future development trends and challenges.

**Table 1:**
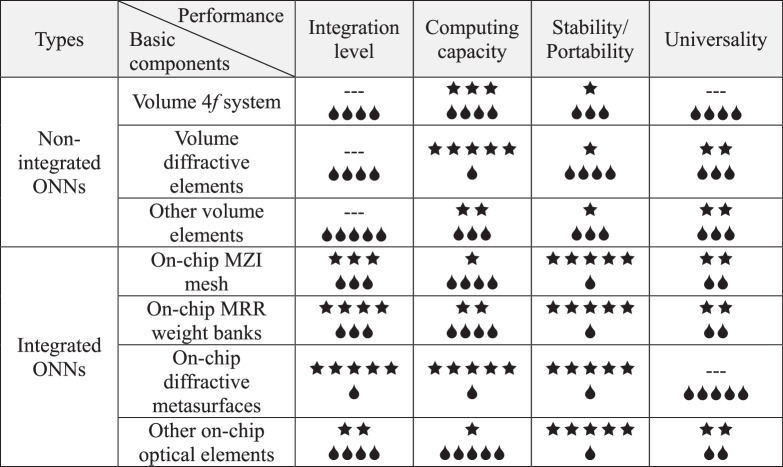
Performance comparison of the non-integrated/integrated ONNs (I)

**Table 2:**
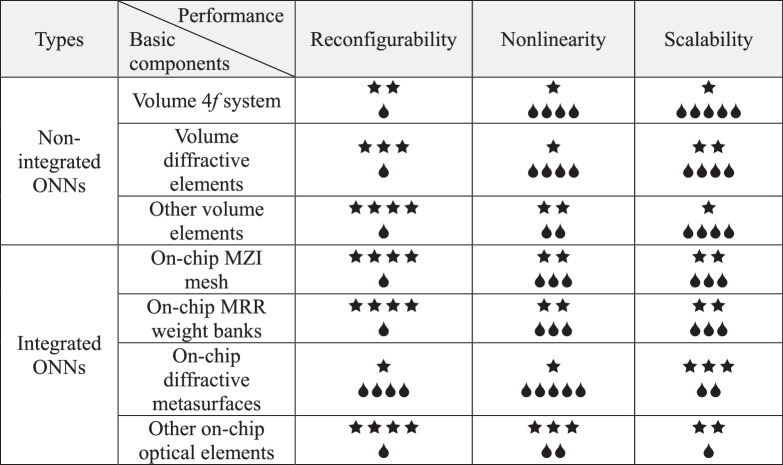
Performance comparison of the non-integrated/integrated ONNs (II)

In Table 1 and Table 2, the number of “” represents the degree to which the corresponding performance of ONNs has been achieved presently. The more “”, the better the performance (the best performance is five “”), and vice versa. The number of “” represents the degree of difficulty that ONNs may achieve the corresponding excellent performance in the future. The more “”, the more difficult it is to achieve the corresponding performance, and vice versa. Meanwhile, five “” indicates that the corresponding performance is the most difficult to implement. In Table 1, the integration level refers to the number of computing units of ONNs that can integrate per unit area. Computing capacity refers to the maximum matrix dimension that ONNs built on different optical components can handle at one time

Non-integrated ONNs are mainly designed based on 4*f* systems^37,39,40^, diffractive elements^42,44,45,53^, and other bulk optical components^62,68,71,73^. All types of the ONNs can achieve reconfigurable functions in terms of technology. Meanwhile, ONNs based on diffractive elements can achieve large computing capacity^42,47^, but it is composed of discrete components, which may cause unavoidable errors in alignment calibration between discrete components. The error will accumulate with the increase of ONN layers, and the accumulated errors will cause adverse impacts on the performance of ONNs. In addition, the ONNs consist of other bulk optical components can already implement nonlinear functions in the optical domain^39,141^, but those ONNs are tough to realize large-scale implementation, which limits their scalability and makes them difficult to achieve universality. In summary, non-integrated ONNs are more suitable for specialized applications, such as specific holographic imaging^142,143^, pre-sensing optical calculation^52,109,112,144^, etc.

For integrated ONNs, thanks to the CMOS process technology, the problem of alignment errors between discrete components have been efficiently solved, and the large-scale and low-cost processing conditions make the scalability and versatility of the integrated ONNs realistic, these advantages effectively make up for the shortcomings of non-integrated ONNs. Among them, the integration level of the ONNs designed based on MZI mesh^60,83,90^, MRR weight banks^98,100,103^, and other components^129,134,138^ is relatively high. However, since these ONNs require constant energy supplies during the working process, which leads to the limitation of large-scale expansion of the computing units by taking into account the adjacent modulator’s thermal crosstalk, thus the computing capacity of these ONNs always keeps a low level. By contrast, the integrated ONNs based on diffractive metasurfaces^110,115^ have a large computing capacity because of the sub-wavelength computing units. However, due to the small size of the computing units (e.g., the size of the diffractive computing unit proposed by Fu et al.^115^ is about 0.5μm×2μm), it is tough to be precisely modulated. Currently, although integrated ONNs have corresponding implementation technologies in terms of reconfigurability^83,90,103,134,138^, nonlinearity^96,145^, and high computing capacity^110,115^, it is still very difficult to simultaneously achieve these advantages on the same ONN, and further exploration is still needed.

### Computational density and computing capacity of ONNs

The computational density in this paper refers to the number of operations that can be completed per square millimeter/centimeter per second. The computing capacity means the maximum matrix dimension that ONNs can handle at one time. Thus, the computational density and computing capacity here are different, in other words, when the computing capacity is large, the computational density may not be high (e.g., the ONN proposed by Lin et al.^42^). In fact, the computational density is significantly related to the integration level of the neurons (computing units) of the ONNs, and the higher the integration level of the computing units of ONNs, the higher the computational density of the ONNs will be^109,110,112,115^. Therefore, the computational density of the integrated ONNs is often higher than that of the non-integrated ONNs. However, in terms of computing capacity, even if the integration level of integrated ONNs is higher than that of non-integrated ONNs, the integrated ONNs may not necessarily have greater computing capacity than non-integrated ONNs.

In addition, regarding the computing capacity of ONNs constructed based on the 4*f* system^37^, we consider that it is related to the integration level of the mask (computing/modulation units are always fabricated on the mask) placed on the Fourier plane. The higher the integration level of the mask on the Fourier plane, the higher the computing capacity of the ONNs.

Finally, in the design of ONNs, it is unnecessary to pursue computational density perversely. It is better to design the ONNs by determining the actual demands, some ONNs with appropriate computing capacity but not high computational density, might also serve the oriented tasks well.

### Nonlinearity and reconfigurability of ONNs

Nowadays, there are cases where both non-integrated ONNs^39,62,71^ and integrated ONNs^96,98,116,146^ can achieve nonlinearity. However, most ONNs that can achieve nonlinearity are generally relatively small in scale. Larger scale ONNs, such as those with larger depths, must consider the optical power attenuation introduced by nonlinear layers.

Regarding reconfigurability, non-integrated ONNs are usually built through optical devices such as SLMs and DMDs^39,40,47^. However, it is tough to achieve optical nonlinearity on these devices. In the future, metasurfaces may be able to simultaneously address issues such as reconfigurability, nonlinearity, and the insertion loss of ONNs. For example, the ONNs designed based on information metasurfaces by Liu et al.^53^ can conveniently achieve the programmable function of each diffractive unit (neuron), and it is promising to further achieve nonlinearity and power amplification function by introducing stable nonlinear amplifiers into artificial neurons^53^.

Integrated ONNs are used to be constructed through optical components such as on-chip MZIs, MRRs, thermal/electro-optical phase shifters, and attenuators^83,96,133,134^. The best performance for integrated ONNs is to achieve both nonlinearity and reconfigurability simultaneously. In fact, the adoption of nonvolatile PCM units^147–150^ can endow ONNs with both reconfigurable and nonlinear functions during the inferring process^96,98^. However, the introduction of new materials for PCM still faces the challenge of insertion loss. Thus, it is imperative to develop new devices that can achieve both reconfigurable and nonlinear functions at low power consumption. Fortunately, Zhong et al.^145^ designed a low power and reconfigurable phase-relevant on-chip activation function device based on Graphene/Silicon heterojunction, which offers a new insight into the on-chip ONNs. The activation function device proposed by Zhong et al.^145^ may be applied to various on-chip ONNs with different architectures in the future.

### Scalability of ONNs

The scalability of non-integrated ONNs is not as good as that of integrated ONNs because of their large size and composition of discrete devices. The scalability of integrated ONNs can be discussed from the following aspects.

First, limitations on the parallel input dimension of signals. In fact, except for integrated ONNs^109,112^ designed based on CMOS chip substrate that are not limited by input dimensions, other integrated ONNs are extremely low (e.g., the input waveguide of less than 10) in the input dimensions due to the limitation of the number of inputting on-chip waveguides^83,90,115,119^.

Second, the scalability of computing units in a single integrated ONN. The on-chip ONNs constructed based on MZI mesh or MRR weight banks require additional energy supply during the operation of the computing units, and many computing units face difficulties in synchronous modulation under high-speed situations. These challenges greatly hinder the large-scale expansion of such on-chip ONNs. Distinctively, due to the computing unit of on-chip DONN consisting of subwavelength diffractive structures^110,115^, its scalability is superior to other on-chip ONNs. The large-scale expansion of on-chip DONN computing units will not cause a significant increase in computing energy consumption, which is advantageous to expand in scale. However, the computing units of on-chip DONN are difficult to achieve reconfigurable and nonlinear functions.

Third, the scalability of cascading on-chip ONNs. At present, all on-chip ONNs have not achieved good scalability in the cascading method. If we hope to further improve the scalability between different on-chip ONNs, electronic circuits may be essential to assist in implementation. Besides, ensuring the supplementation or regeneration^53^ of optical power during the light propagation in cascaded ONNs is crucial. Optoelectronic hybrid ONNs^47^ may be an effective way to achieve cascade scalability between various on-chip ONNs. However, the energy consumption caused by the photoelectric conversion interface and the impact of conversion speed on the overall efficiency of the optoelectronic hybrid ONN system will become undeniable. Notably, scalabilities between the on-chip ONNs require the support of nonlinear functionality, otherwise, the performance improvement of the scalable ONNs will be compromised. Thus, there are still existing many challenges in the scalable process of ONNs.

### Energy efficiency of ONNs

The calculation process of ONNs is completed during the propagation of light, thus their energy efficiency during operation can be designed to be excellent. Here we provide quantitative comparisons of the energy efficiency between different ONNs and compare it with the energy efficiency of the advanced computing hardware, as shown in Table 3. It is not difficult to find that the energy efficiency of some ONNs^83,134^ is better than that of existing advanced computing hardware^151,152^, and the advantage in energy efficiency will be extremely prominent^153^ after optimizing the on-chip ONN’s architecture. For more performance summaries of ONNs, including energy efficiency and other performance aspects please refer to more relevant reference works^32,47,110,115,153–156^.

**Table 3:** Energy efficiency comparison of partial different optical computing architecture and electronic hardware

Works	Technology	Theoretical energy efficiency (TOPS/W)
Ref. ^47^	MPLC with a reconfigurable DPU	0.71
Ref. ^83^	Coherent MZI mesh	6.67
Ref. ^98^	Photonic in-memory computing based on WDM system and PCM units	0.50
Ref. ^134^	Based on integrated attenuators	2.90
Ref. ^153^	Based on integrated subwavelength diffractive units and MZI mesh	160.82
Ref. ^151^	HUAWEI Ascend 910	1.65
Ref. ^152^	NVIDIA Tesla T4	1.85
Ref. ^160^	Google TPU	4.65

In Table 3, the abbreviations are *MPLC* multiple plane light conversion, *DPU* diffractive processing unit, *TPU* tensor processing unit, and *PCM* phase change material. Indicates that the modulation and detection rates are set at 10 GHz during calculation

### Applications of ONNs

Currently, the application of ONNs is not as widespread as its electronic counterparts, most research works still focus on handling simple datasets, and the practical application scenarios of ONNs are rare. For all that, scientific researchers are striving to combine the research study of ONNs with real-world applications. For example, Huang et al.^99^ applied on-chip integrated ONNs to nonlinear compensation in submarine fiber optic communication links. Sludds et al.^157^ developed the Netcast, an edge-computing architecture based on photonic deep learning, to complete the inferring process on edge devices. The dedicated ONNs in real-world scenarios have indeed achieved positive results, bringing benefits to practical application systems regarding computing speed, energy consumption, etc.

In addition, the industry has also invested in research in the field of ONNs or optical computing. For example, Lightmatter has successively released a series of products such as Envise and Passage^158^. The company has comprehensively considered software and hardware collaboration and energy consumption to better leverage the inherent advantages of light in the optical computing process. Lightelligence has released the photonic arithmetic computing engine (PACE), which integrates more than 10,000 discrete photonic devices in PACE’s photonic chip and has a system clock of 1 GHz^159^.

**Fig. 17:**
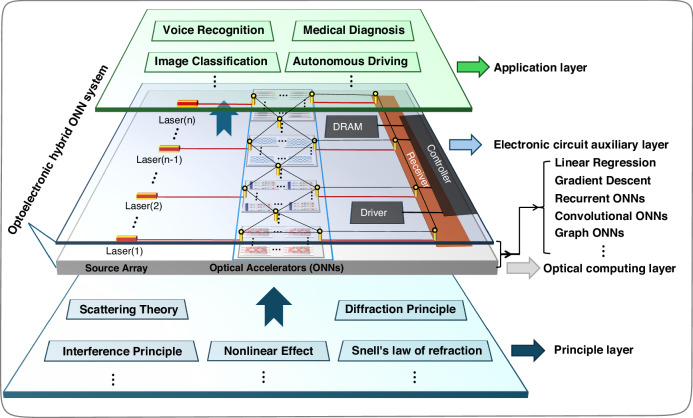
**Design principles of ONNs and its optoelectronic hybrid system**. Schematic diagram of the optoelectronic hybrid ONNs system framework, including the principle layer, optical computing layer, electronic circuit auxiliary layer, and application layer

Admittedly, ONNs are difficult to independently complete inference tasks without the assistance of electronic computing hardware, and the application of using ONNs to thoroughly replace the electronic counterpart is still far away. The universality performance of all ONNs as shown in Table 2 is relatively low, which indicates that the application fields of ONNs are not broad. Here, we introduce an optoelectronic hybrid framework in the application of ONNs. In the framework, ONNs are the optical accelerators as part of the optoelectronic hybrid system, as shown in Fig. 17.

Among the optoelectronic hybrid ONNs system framework, the main task of ONNs is to efficiently process a host number of linear matrix operations at the speed of light and undertake the main computational work in optoelectronic hybrid systems. Meanwhile, the responsibility of electronic auxiliary hardware is to achieve parameter reconstruction of ONNs and handle nonlinear operations, data storage, and flow control that are difficult to implement by ONNs. By combining the advantages of electronic hardware and ONNs, the performance of an optoelectronic hybrid system will be superior to traditional electronic methods in terms of energy consumption, computing capacity, computing speed, and so forth^83,115,134,153^. It is worth noting that when processing massive amounts of data, there will be a host number of routing operations and optoelectronic (electro-optic) conversion operations in the calculation process of optoelectronic hybrid systems. Therefore, new optoelectronic communication protocols and optoelectronic (electro-optic) conversion efficiency as well need to be further optimized.

**Fig. 18:**
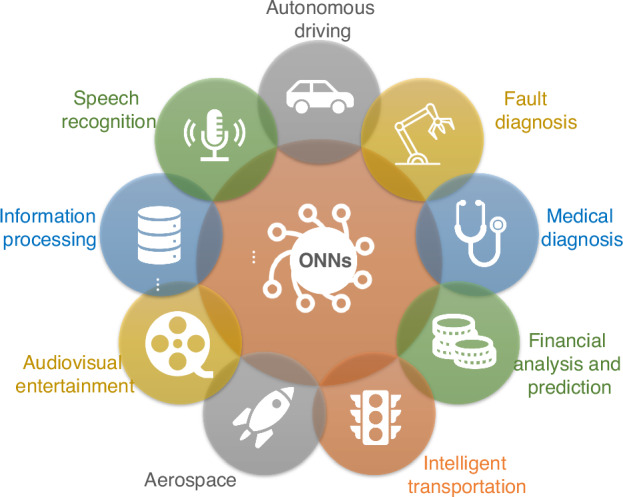
**Application of future ONNs in various fields**. ONNs will achieve better performance than their electronic counterparts in various specialized fields such as information processing, medical diagnosis, aerospace, etc

In the future, it may take a considerable period to continuously optimize the architecture or hybrid framework of the ONNs system to obtain better performances, so that they can achieve outstanding results in certain dedicated fields (Fig. 18) compared to their electronic counterparts. During this period, there may be challenges in comprehensively considering the construction of the ONNs application ecosystem, including software, hardware, protocols, optical algorithms, industry standards, manufacturing technology, and other aspects.

## Conclusion

In this review, we first introduce the development history of ONNs, the mathematical model of optical artificial neurons, and the distinctions between various optical components to achieve optical matrix operations. Secondly, we systematically retrospect the development of ONNs based on seven diverse optical components of non-integrated ONNs and integrated ONNs and introduce the typical research works in detail. Then, we score and analyze the performances of different types of ONNs in the discussion part, including computational density, computing capacity, reconfigurability, nonlinearity, and scalability. Finally, we discuss the challenges that the various ONNs may encounter and envisage their applications and development trends in the future.
